# Prospective birth cohort: Cephalosporins drive gut microbiota and food allergy^☆^

**DOI:** 10.1016/j.waojou.2026.101475

**Published:** 2026-10-08

**Authors:** Ning Zhu, Yimin Zhao, Fangyuan Cai, Huixi Tu, Zhangye Xu, Yuexin Gu, Xiaoye Lv, Lei Wang, Weixi Zhang

**Affiliations:** a Department of Pediatric Allergy and Immunology, The Second Affiliated Hospital and Yuying Children's Hospital of Wenzhou Medical University, Wenzhou, China; b Department of Obstetrical, The Second Affiliated Hospital and Yuying Children's Hospital of Wenzhou Medical University, Wenzhou, China; c The Second Clinical Medical College of Wenzhou Medical University, Wenzhou, China

**Keywords:** Cephalosporin antibiotics, Food allergy, Gut microbiota, Metabolite, Prospective cohort

## Abstract

**Background:**

The biological association between early-life antibiotic exposure and the risk of food allergy (FA) remains unclear. Given the widespread use of cephalosporins during the perinatal period and infancy, this relationship and potential mechanisms urgently require clarification.

**Methods:**

This study employed a prospective birth cohort design, recruiting 1275 mother-infant pairs, followed until 6 months of age, recorded cephalosporins exposure timing, and collected fecal samples for multi-omics analysis.

**Results:**

Nine hundred thirty-nine eligible mother-infant pairs completed the study, and 224 infants were diagnosed with IgE-FA. Multivariate analysis identified intrapartum cephalosporins exposure duration (OR = 1.04) and the first 2 months of life (0–1 months of age: OR = 4.03; 1–2 months of age: OR = 3.13) as critical risk windows for cephalosporins exposure-induced FA. Family allergic history was a synergistic risk factor. This effect exhibited an “all-or-none” pattern, independent of antibiotic dose or treatment course, suggesting it may act as a “switch” triggering allergic susceptibility. Gut microbiota and metabolomic analyses revealed that cephalosporins exposure led to dysbiosis, downregulation of tryptophan metabolism and nitric oxide (NO) production pathways, which were associated with impaired intestinal barrier function and T-helper 2 (Th2) immune imbalance.

**Conclusions:**

This study is the first to precisely delineate the critical temporal windows of early-life cephalosporins exposure in the development of IgE-FA, propose the “switch hypothesis”, and preliminarily explore its underlying mechanisms. These findings provide a novel rationale for optimizing the precise use of antibiotics during the intrapartum period and infancy, and for FA interventions targeting the microbial metabolic axis.

**Trial registration number:**

ChiCTR2300072832.

## Graphical abstract

**Figure:**
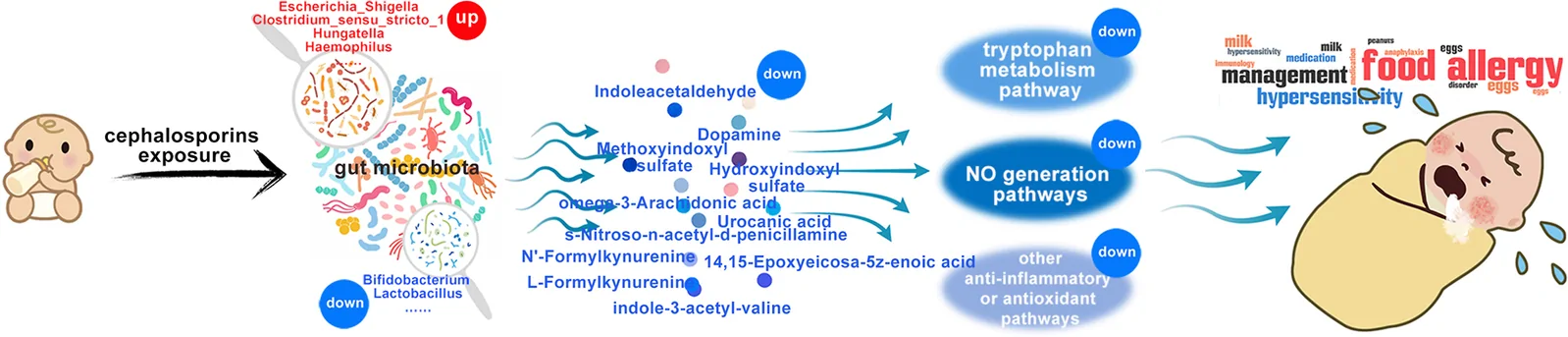

**Key messages:**

**Brief summary:** Through a prospective birth cohort, we established the association of cephalosporin use with FA and uncovered its potential mechanisms.

**What is already known on this topic?**

Food allergy (FA) rates are rising, but the link between early-life antibiotics and infant FA is unclear. Given the widespread use of cephalosporins, this relationship and mechanisms worth exploring.

**What this study adds?**

This study is the first to pinpoint critical early-life windows for cephalosporins exposure in IgE-FA development, propose the “switch hypothesis”, and explore its mechanisms.

**How this study might affect research, practice or policy?**

These findings provide a novel rationale for optimizing the precise use of antibiotics during the intrapartum period and infancy, and for FA interventions targeting the microbial metabolic axis.

## Introduction

Food allergy (FA), recognized as the second wave of the allergy epidemic,^1^ is a growing global public health concern, affecting about 8% of children in developed countries.^2^ The estimated national prevalence of FA in China ranges from 3.13% to 11.9% in northern regions and 3.5%–21.13% in the south,^3^ exhibiting a notable north-south gradient. The allergy rate in coastal cities is generally relatively higher, with reported rates of 7.0% in Guangzhou, 16.6% in Hong Kong, 16.87% in Jiangsu, and 18.99% in Wenzhou.^3^, ^4^, ^5^, ^6^ However, the true prevalence of infant FA remains unclear. Official statistics are likely underestimated due to nonspecific symptoms, access barriers, and natural tolerance development. Of note, this uncertainty is compounded by the potential for over-reporting by caregivers, highlighting the need for non invasive biological testing.

Evidence linking antibiotic exposure, whether during the peri-partum period or infancy, to FA risk remains inconsistent.^7^ The study by Mai Ofri et al demonstrated that systemic antibiotic exposure during the neonatal period was associated with a threefold increased risk of FA up to age 6 (OR = 2.89, 95% CI = 1.34–6.92, P = 0.01),^8^ yet this was not corroborated by a subsequent population-based cohort study from South Korea after controlling for confounders.^9^ Likewise, the association with early-life postnatal exposure remains unresolved.^10^^,^^11^ These inconsistencies may largely stem from methodological heterogeneity, particularly the conflation of diverse antibiotic categories and the use of broad, non-specific exposure windows. Different antibiotic categories exert distinct effects on the infant gut microbiota which serves as a critical regulator of immune system maturation. The work of Reyman et al work revealed the amoxicillin + cefotaxime regimen showed the most outspoken ecological effects, especially on overall microbial community composition and stability of microbiota development, while the co-amoxiclav + gentamicin regimen showed the most obvious reduction in microbial diversity.^12^ Consequently, pooling them into a single exposure variable could dilute or obscure risk signals specific to certain antibiotics. Especially cephalosporins, as the preferred prophylactic medication in obstetrics,^13^ represent a near-universal exposure in specific clinical contexts, however no study to date has specifically investigated the long-term allergic outcomes associated with this prevalent exposure. To address this gap and eliminate confounding by non-cephalosporins, we focus specifically on cephalosporins exposure during the perinatal period. As for the determination of the infancy antibiotic exposure type, due to the lack of epidemiological statistics on antibiotic prescriptions for Chinese infants, all types of infancy antibiotics exposure records were included during the initial data collection. Besides, to move beyond simple association, we collect infants’ feces at 6 months of age^14^ to explore its potential mechanisms.

By precisely defining the exposure factors and integrating mechanistic insights, this prospective birth cohort study aims to clarify the link between early-life cephalosporins exposure and FA, explore potential micro mechanisms.

## Methodology

### Participants

This prospective study enrolled 1275 eligible mother-infant dyads from our hospital between May 2023 and December 2024. The study protocol was approved by the Hospital Ethics Committee (No. 2022-K-339-01) and the reporting adhered to the STROBE guidelines,^15^ with a completed checklist available in Supplementary Materials A.

Inclusion criteria: 1) Mothers were non-smokers without severe obstetric complications; 2) Both parents were free of active infectious diseases and had no family history of hereditary disorders; 3) Both parents agree to participate and are willing to conduct one-year follow-up (signing an informed consent form).

Exclusion criteria: 1) mother used any antibiotics during pregnancy; 2) maternal administration of non-cephalosporin antibiotics during delivery; 3) missing baseline clinical data; 4) infants suffer from serious diseases such as congenital heart disease, bronchopulmonary dysplasia, congenital metabolic disorders, immunological diseases, or life-threatening comorbidities, etc.

A multimodal follow-up system was established utilizing instant messaging app, free outpatient interviews, and face-to-face evaluations to reduce missing rate.

#### Materials collection

We systematically collected a comprehensive dataset encompassing maternal, neonatal, family, and follow-up parameters to evaluate early-life FA risk. Maternal data included demographic characteristics, prenatal ultrasound parameters, anthropometrics, obstetric complications, reproductive history, delivery details, and intrapartum antibiotic exposure. Neonatal data comprised birth weight and length. Family history included documented allergic diseases. Follow-up data at 6 months covered infant feeding pattern, antibiotic exposure, allergic symptoms assessed by World Health Organization (WHO) criteria, and fecal specimen collection. Medication records were verified via China's national medical insurance system to ensure accuracy.

### Food allergy diagnosis

To establish an Immunoglobulin E mediated FA (IgE-FA) diagnosis, a 4-week dietary elimination of the suspected allergens (reported by parents) was implemented under the supervision of specialists.^16^ Breastfeeding mothers should also avoid certain foods. Eligibility was defined as meeting the following criteria during the subsequent food challenge: 1) a clear reduction in the scope and severity of allergic symptoms (mainly include: repeated running nose, cough, wheeze, diarrhea, vomit, rash, crying and restless, feeding difficulty, slow weight gain, slow height gain, and somnipathy) during the elimination period, followed by symptoms recurrence upon re-exposure; and 2) any specific IgE (sIgE) concentration ≥0.35 kUA/L.^17^ The diagnostic procedure will be carried out promptly whenever a child presents with suspected symptoms. All enrolled children are scheduled to complete the evaluation by the age of 6 months at the latest.

### Antibiotic exposure window

In accordance with the standard clinical practice in China where antibiotic administration in pregnant women is typically confined to the 24-h period after the onset of labor,^13^^,^^18^ we defined intrapartum antibiotic exposure as the administration within the 24 h preceding fetal delivery. Early-life gut colonization profoundly shapes the subsequent development of the microbiota, and perturbations during this critical window may have long-lasting effects.^19^ Therefore, we subdivided the infancy antibiotic exposure time window into 0–1, 1–2, and 2–6 months of age.^20^

### 16S rRNA amplicon sequencing

Donated fecal samples were stored at −80 °C. To mitigate batch effects, all samples were sequenced simultaneously. Bacterial DNA was selectively amplified via polymerase chain reaction (PCR) targeting the conserved region of the 16 S rRNA gene, followed by agarose gel electrophoresis and purification. Sequencing of the purified products enabled identification of bacterial communities and comprehensive assessment of gut microbiota diversity.

### Non-targeted global metabolomics analysis

A non-targeted global metabolomic analysis was conducted using liquid chromatography-tandem mass spectrometry (LC-MS/MS) technology. The fecal samples were prepared through appropriate extraction techniques and filtered before analysis. The analysis process was carried out using Progenesis QI v3.0 software.

### KEGG pathway enrichment analysis

To investigate the biological pathways associated with the target metabolomic set, we performed pathway enrichment analysis based on the Kyoto Encyclopedia of Genes and Genomes (KEGG) database. The target metabolomic set was first mapped to the KEGG database to obtain pathway annotations. Subsequently, a hypergeometric test was applied to evaluate the significance of enrichment for each KEGG pathway within the target metabolomic set. The resulting P-values were corrected using the Benjamini-Hochberg method to control the false discovery rate, and pathways with an adjusted P-value <0.05 were considered significantly enriched.

### Statistical analyses

All analyses were performed using IBM SPSS Statistics 26 and R 4.5.2. Group differences were assessed with t-tests or one-way ANOVA for normally distributed data, and the Mann-Whitney *U* test for non-parametric data. Categorical variables were compared using Pearson's chi-square and Kruskal-Wallis tests. Independent risk factors were identified via multivariate logistic regression (threshold P < 0.05). Correlations were evaluated using Spearman's rank correlation. The microbial and metabolite samples were initially selected using R software, with the aim of extracting the maximum comparable sample size under controlled confounding variables (P > 0.05), prior to subsequent analyses. A random forest algorithm (seed = 123) was employed for sampling. Statistical significance was set at P < 0.05.

## Results

### Demographic characteristics

This study included 1275 mother-infant dyads. By the end of follow-up, 1052 dyads had completed the study. The attrition rate was 17.5% (n = 223), primarily due to refusal to undergo IgE-FA diagnosis or losing contact. Ultimately, 224 children were confirmed with IgE-FA (17.57%). The distribution of confirmed food allergens was dominated by cow's milk (n = 185), egg (n = 159), shrimp (n = 20), and crab (n = 15). Other identified allergens included nuts (n = 11), fish (n = 10), wheat (n = 6), and soybeans (n = 4). Less prevalent allergens (n ≤ 3) comprised beef, leafy greens, mushrooms, peaches, and taro. Among the participants, 69 (30.8%) presented with a single allergy, 129 (57.6%) had 2 concurrent allergies, 16 (7.1%) had 3 concurrent allergies, and 10 (4.5%) had 4 or more concurrent allergies.

### Risk factors for FA

Baseline data from 1052 mother-infant dyads with completed follow-up were verified. During the follow-up period, 542 infants were prescribed cephalosporin antibiotics, while 51 infants received non-cephalosporin antibiotics (28 infants used penicillin, 22 used azithromycin, and 1 used other antibiotics). This indicates cephalosporins are predominantly prescribed in Chinese infant antibiotic treatments, and therefore the postnatal exposure component of this study focused on the impact of early-life cephalosporins exposure on infant IgE-FA. After excluding 51 infants who used non-cephalosporin antibiotics, ultimately, 939 pairs were included in the analysis (participation process is shown in Fig. 1). Other exclusions (n = 62) were due to: 25 mothers retrospectively found to have received antibiotic prescriptions during pregnancy, 21 mothers unable to provide valid early pregnancy records, 13 mothers with retrospectively diagnosed preeclampsia, and 3 infants diagnosed with growth retardation. The demographic baseline characteristics are shown in Table 1.

**Fig. 1:**
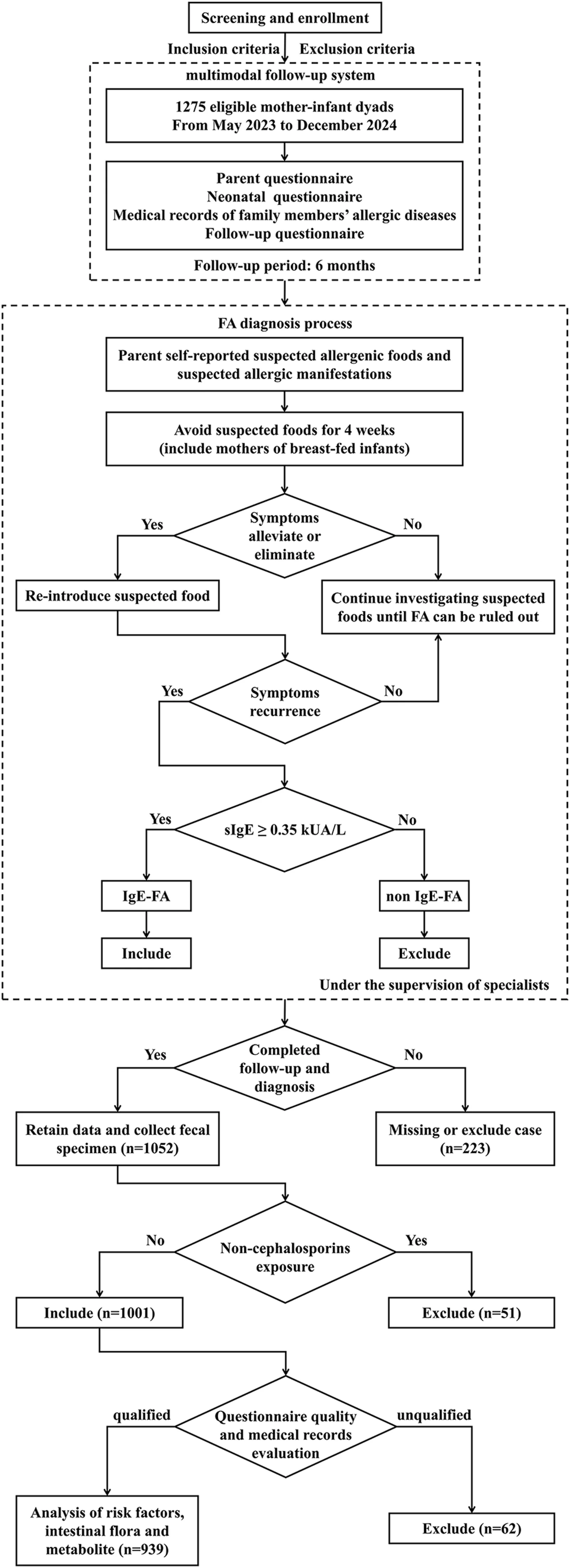
**Flowchart of participant enrollment and follow-up.** FA, food allergy. sIgE, specific immunoglobulin E. IgE-FA, Immunoglobulin E mediated FA. Values represent the number of participants (n) at each stage.

**Table 1:** Demographics of participants.

Level	Healthy children (n = 715)	FA children (n = 224)	P-value	
Single factor intergroup comparison				
Maternal characteristics				
Maternal pre pregnancy weight (kg)	54.2 [49.0, 58.0]	54.1 [48.0, 58.5]	0.776	
Maternal height (cm)	160.6 [158.0, 164.0]	160.9 [158.0, 164.0]	0.628	
Maternal pre pregnancy BMI (kg/m^2^)	21.0 [19.07, 22.41]	20.8 [18.75, 22.33]	0.524	
Maternal prenatal weight (kg)	68.0 [61.0, 73.0]	68.1 [62.0, 73.1]	0.798	
Maternal prenatal BMI (kg/m^2^)	26.3 [23.93, 28.28]	26.3 [24.25, 28.23]	0.731	
Delivery age (year)	29.5 [27.0, 32.0]	29.5 [27.0, 32.0]	0.870	
Hepatitis B virus (HBV) infection (%)			0.388	
None	691 (96.6)	219 (97.6)		
HBeAg-negative chronic HBV infection	18 (2.6)	5 (2.4)		
HBeAg-positive chronic HBV infection	6 (0.8)	0 (0.0)		
Anemia during pregnancy (%)			0.786	
No	660 (92.3)	208 (92.8)		
Yes	55 (7.7)	16 (7.2)		
Streptococcus agalactiae (%)			0.981	
Negative	670 (93.7)	212 (93.4)		
Positive	45 (6.3)	15 (6.6)		
Placenta previa (%)			0.965	
No	706 (98.8)	222 (99.1)		
Yes	9 (1.2)	2 (0.9)		
Premature rupture of membranes (%)			0.657	
No	564 (78.8)	177 (79.0)		
Yes	151 (21.2)	47 (21.0)		
Nuchal cord (%)			0.658	
No	468 (65.4)	143 (63.8)		
Yes	247 (34.5)	82 (36.2)		
Delivery mode (%)			0.886	
Vaginal delivery	492 (68.8)	153 (68.3)		
Cesarean section	223 (31.2)	71 (31.7)		
Gravidity	1.8 [1.0, 2.0]	1.7 [1.0, 2.0]	0.227	
Parity	1.3 [1.0, 2.0]	1.3 [1.0, 2.0]	0.162	
Infant characteristics				
Gender (%)			0.041	
Male	359 (50.3)	130 (58.0)		
Female	356 (49.7)	94 (42.0)		
Fetal gestational age (week)	38.8 [38, 39]	38.9 [38, 40]	0.054	
Birth length (cm)	49.75 [49, 50]	49.91 [50, 50]	0.144	
Birth weight (g)	3267.39 [3005, 3510]	3305.09 [3077.5, 3542.5]	0.214	
Family allergic history			0.001	
Yes	480 (67.1)	100 (44.4)		
No	235 (32.9)	124 (55.1)		
Feeding practice			0.092	
Exclusive breastfeeding	137 (19.1)	54 (24.1)		
Breastfeeding primarily	104 (14.5)	35 (15.6)		
Mixed feeding	301 (42.0)	86 (38.3)		
Formula feeding primarily	100 (13.9)	29 (14.7)		
Exclusive formula feeding	73 (10.2)	12 (7.1)		
Age for introducing dairy products			0.562	
No	140 (19.6)	47 (21.0)		
0–3 months of age	498 (69.7)	143 (63.8)		
4–5 months of age	50 (7.0)	26 (11.6)		
6 months of age	27 (3.8)	8 (3.6)		
Age for introducing juice			0.476	
No	647 (90.5)	199 (88.8)		
0–3 months of age	1 (0.1)	0		
4–5 months of age	22 (3.1)	9 (4.0)		
6 months of age	45 (6.3)	16 (7.1)		
Age for introducing solid complementary food			0.069	
3 months of age	1 (0.1)	0		
4 months of age	3 (0.4)	2 (0.9)		
5 months of age	182 (25.5)	66 (29.5)		
6 months of age	349 (48.8)	112 (50.0)		
No	180 (25.2)	44 (19.6)		
Age for introducing grain			0.431	
0–3 months of age	1 (0.1)	0		
4–5 months of age	70 (9.8)	28 (12.5)		
6 months of age	197 (27.6)	64 (28.6)		
No	447 (62.5)	132 (58.9)		
Age for introducing vegetable			0.989	
0–3 months of age	1 (0.1)	0		
4–5 months of age	56 (7.8)	22 (9.8)		
6 months of age	116 (16.2)	33 (14.7)		
No	542 (75.8)	169 (75.4)		
Age for introducing fruit			0.926	
0–3 months of age	0	0		
4–5 months of age	45 (6.3)	15 (6.7)		
6 months of age	116 (16.2)	35 (15.6)		
No	554 (77.5)	174 (77.7)		
Age for introducing egg			0.486	
0–3 months of age	0	0		
4–5 months of age	15 (2.1)	3 (1.3)		
6 months of age	152 (21.3)	44 (19.6)		
No	548 (76.6)	177 (79.0)		
Age for introducing meat			0.621	
0–3 months of age	0	0		
4–5 months of age	8 (1.1)	5 (2.2)		
6 months of age	78 (10.9)	25 (11.2)		
No	629 (88.0)	194 (86.6)		
Age for introducing seafood			0.218	
0–3 months of age	0	0		
4–5 months of age	4 (0.6)	2 (8.9)		
6 months of age	29 (4.1)	13 (5.8)		
No	682 (95.4)	209 (93.3)		
Cephalosporins exposure episodes	0.60 (0,1)	0.80 (0,1)	0.001	
Exposure duration in parturition (h)	1.48 (0, 0.82)	2.23 (0, 1.14)	0.016	
Exposure within 0–1 months of age	31 (4.3)	38 (17.0)	<0.001	
Exposure within 1–2 months of age	15 (2.1)	12 (5.3)	0.011	
Exposure within 2–6 months of age	57 (8.0)	17 (7.6)	0.853	
Reasons for cephalosporins exposure				
Maternal parturition exposure	327	108	0.347	
Fever	26	16	0.329	
Cough	46	24	0.638	
Vomit	2	1	0.489	
Diarrhea	24	13	0.225	
Other/unknown	33	18	0.347	
Dosage (Unit dose per body weight)	1.03 (1,1)	1.03 (1,1)	0.233	
Course (define 1 course = 3 days)	1.84 (1,2)	1.84 (1,2)	0.987	
Multivariate logistic regression analysis				
	OR	95% CI	P	
Gender	0.76	0.55–1.04	0.082	
Family allergic history	1.57	1.15–2.16	0.005	
Cephalosporins exposure episodes	0.94	0.70–1.27	0.701	
Exposure duration in parturition (h)	1.05	1.01–1.08	0.019	
Exposure within 0–1 months of age	4.28	2.38–7.67	<0.001	
Exposure within 1–2 months of age	3.19	1.34–7.57	0.009	

Note: Continuous variables with non-normal distribution were expressed as average and interquartile range in parenthesis. Categorical data were presented as number and percentages in parenthesis. P > 0.05 indicates that there is no statistical difference between the groups. Variables with a P-value <0.05 in the univariate analysis were entered into the multivariate regression analysis

Through twice multivariate logistic regression, we found family allergic history (OR = 1.58, 95% CI 1.152–2.171), cephalosporins exposure within 0–1 months of age (OR = 4.03, 95% CI 2.404–6.761) and 1–2 months of age (OR = 3.13, 95% CI 1.385–7.051), as well as time of cephalosporins exposure duration during delivery (OR = 1.04, 95% CI 1.008–1.079) were independent risk factors. Neither antibiotic dosage, course, nor total number of exposure episodes demonstrated statistically significant differences. We speculate that this is because a single standard-dose cephalosporin antibiotic exposure is sufficient to reach the maximum pathogenic effect, ie, switch function.

When we analyzed cephalosporins exposure time in combination with positive family allergic history, we found the OR values remain stable and robust. The OR value for antibiotic exposure at 0–1 months of age increased to 12.14 (p < 0.001, 95% CI 5.154–28.58). The time of cephalosporins exposure duration during delivery reached to 1.14 (p < 0.001, 95% CI 1.065–1.209), and with the interval prolongs (ie, the greater the possibility and longer the duration of cephalosporins entering the fetus), the risk gradually increases (shown in Table S1), especially in 12–18 h before fetal expulsion (OR = 4.69, p = 0.044, 95% CI 1.041–21.168). But no statistical significance was observed, which may be attributed to the small sample size of positive cases, especially in 18–24 h group (n = 4).

### Intestinal flora differences

Gut microbiota dysbiosis plays an important role in intestinal immunity and inflammation,^21^ so we hypothesized that cephalosporins contribute to IgE-FA by affecting the gut microbiota. Given that cephalosporins exposure during the 0–1 months of age period had the greatest impact, we included a total of 321 fecal samples for gut microbiota analysis after controlling for confounders (FA group: Control group = 129 : 192). Since the strong association between milk allergy and egg allergy, the children included were diagnosed with milk allergy, either with or without egg allergy. The confounders are listed in Table S2, except for the difference in cephalosporins exposure history at 0–1 months of age, there were no other differences between the 2 groups.

Analysis of 52 absolute microbial abundance revealed that *Bifidobacterium*, *Lactobacillus*, and *Fusicatenibacter* were decreased in the FA group, while *UBA1819* was increased (Fig. 2A). The FA group showed an increase in pathogenic or pro-inflammatory genera such as *Escherichia_Shigella*, *Hungatella*, and *Haemophilus*, whereas the control group was characterized by a predominance of anti-inflammatory or beneficial genera (Fig. 2B). Based on LDA scores and random forest analysis, a receiver operating characteristic (ROC) curve was constructed to predict FA, achieving an accuracy of 0.92 (Fig. 2C), indicating excellent classifier performance. These suggest cephalosporins disturb gut microbiota homeostasis, favoring the proliferation of opportunistic pathogens and pro-inflammatory bacteria.

**Fig. 2:**
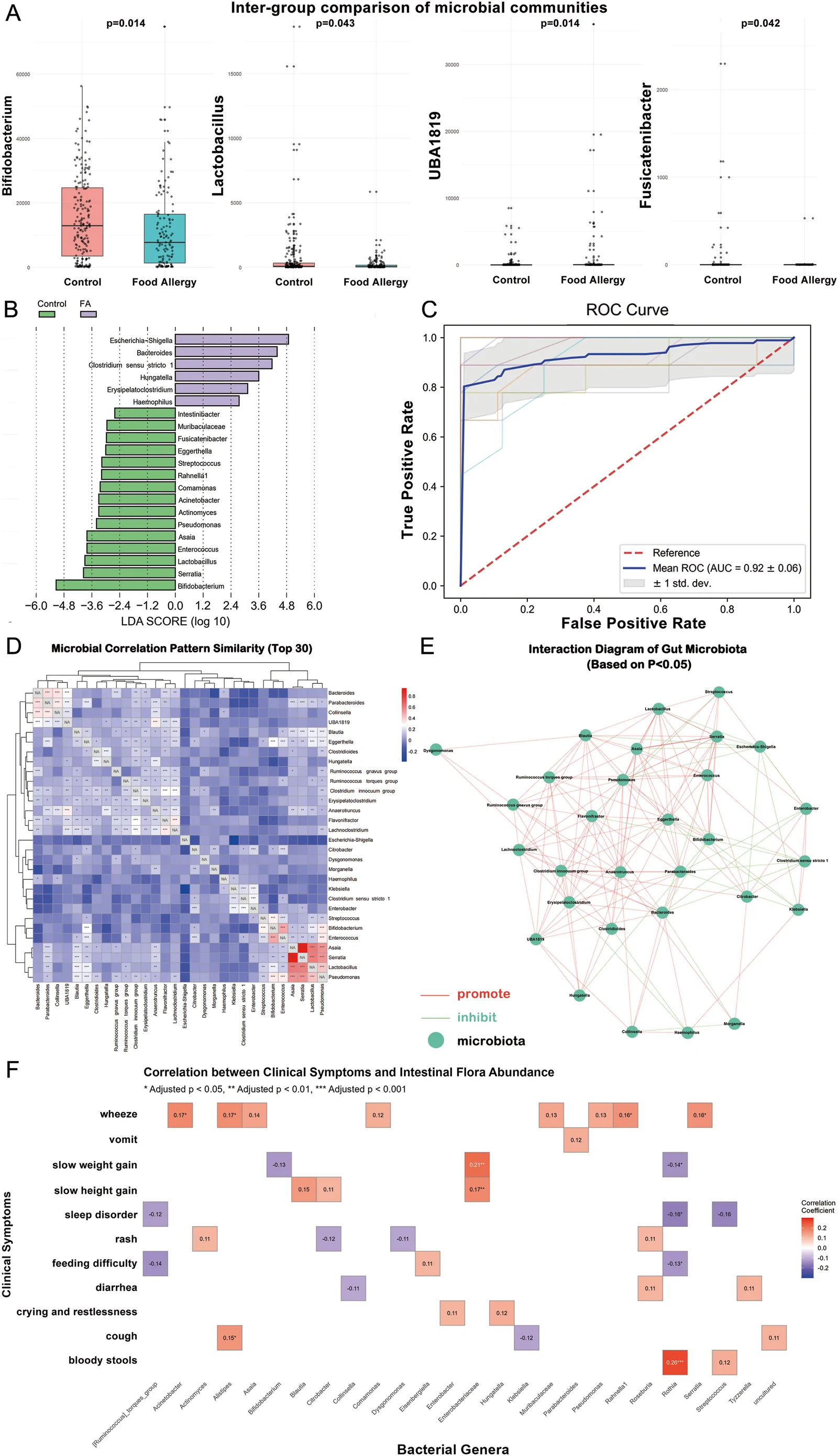
**Gut microbiota comparisons between FA group (n=129) and Control group (n=192). (A)** Analysis of absolute microbial abundance. Compare to Control group, *Bifidobacterium*, *Lactobacillus*, and *Fusicatenibacter* decreased while *UBA1819* increased. Remaining bacterial genera showed no statistical difference. **(B)** LDA score distribution of significantly different bacterial genus between the 2 group. A larger absolute value of the LDA score (after log 10 transformation) indicates a more significant difference. The right-side bars list bacterial genera significantly enriched in FA group and the left-side bars list bacterial genera significantly enriched in Control group. **(C)** The receiver operating characteristic (ROC) curve for distinguishing FA based on bacterial genera. The area Under Curve (AUC) > 0.9 indicates the model has excellent discriminative power. **(D)** Heatmap of Top 30 bacterial genera association based on Spearman's rank correlation analysis. Red indicates positive correlation and blue indicates negative correlation, and lighter colors indicate weaker correlation. ∗ indicates P < 0.05, ∗∗ indicates P < 0.01, ∗∗∗ indicates P < 0.001. Hierarchical clustering grouped microbial genera with similar correlation patterns into distinct branches. **(E)** Interaction diagram of gut microbiota based on P < 0.05. Red line indicates promoting effect and green line indicates inhibitory effect. **(F)** Heatmap of clinical symptoms with bacterial genera. Clinical symptoms include running nose, cough, wheeze, vomit, diarrhea, bloody stools, rash, crying and restlessness, feeding difficulty, slow weight gain, slow height gain, sleep disorder. Due to the excessive amount of raw data, only the patterns with P < 0.05 are shown in the figure. Red indicates positive correlation and blue indicates negative correlation, and lighter colors indicate weaker correlation. ∗ indicates Adjusted P < 0.05, ∗∗ indicates Adjusted P < 0.01, ∗∗∗ indicates Adjusted P < 0.001 (Adjusted P < 0.05 usually indicates a strong association or strong statistical significance).

Correlation analysis at the genus level (Fig. 2D and E) revealed the highly influential microbiota in FA group were predominantly negatively correlated with those in Control group, while strong positive correlations were generally observed within each group. FA symptoms are also correlated with the abundance of key gut microbiota (Fig. 2F). We found that *Rothia* was negatively correlated with sleep disorder, slow weight gain, and feeding difficulty, but positively correlated with bloody stools. *Enterobacteriaceae* showed a positive correlation with poor weight and height gain. Wheeze was positively correlated with various types of bacilli.

### Metabolite differences

Due to budget constraints, we conducted non-targeted metabolite analysis on only half of the samples (FA group: Control group = 87 : 90). The sample extraction was performed by R 4.5.2 and confounders are listed in Table S3.

The metabolites were predominantly enriched in 5 super classes: Organic acids and derivatives, Organoheterocyclic compounds, Lipids and lipid-like molecules, Benzenoids, and Organic oxygen compounds (Fig. 3A). Among the top 1000 metabolites, we found 27 metabolites associated with anti-inflammatory or antioxidant functions (Fig. 3B). Notably, all of them exhibited a down-regulated trend, especially metabolites in the tryptophan metabolic pathway. A heatmap analysis showed that microbiota with higher LDA scores in the FA group were negatively correlated with these metabolites, whereas microbiota with higher LDA scores in the Control group showed positive correlations (Fig. 3C). This pattern further supports the proposed cascade linking cephalosporins exposure to FA through sequential gut microbiota dysbiosis and downregulation of key metabolites. Beyond the findings presented in Fig. 3C, our analysis also revealed that *Clostridium_sensu_stricto_1* showed a negative correlation with Hydroxyphenyllactic acid (*r* = −0.30, Adjusted P = 0.012), which is a fermentation product of *Lactobacillus* with antioxidant and anti-inflammatory properties. *Erysipelatoclostridium* was positively correlated with Azelaic acid (*r* = 0.26, Adjusted P = 0.026), suggesting an elevated level of oxidative stress in FA infants.

**Fig. 3:**
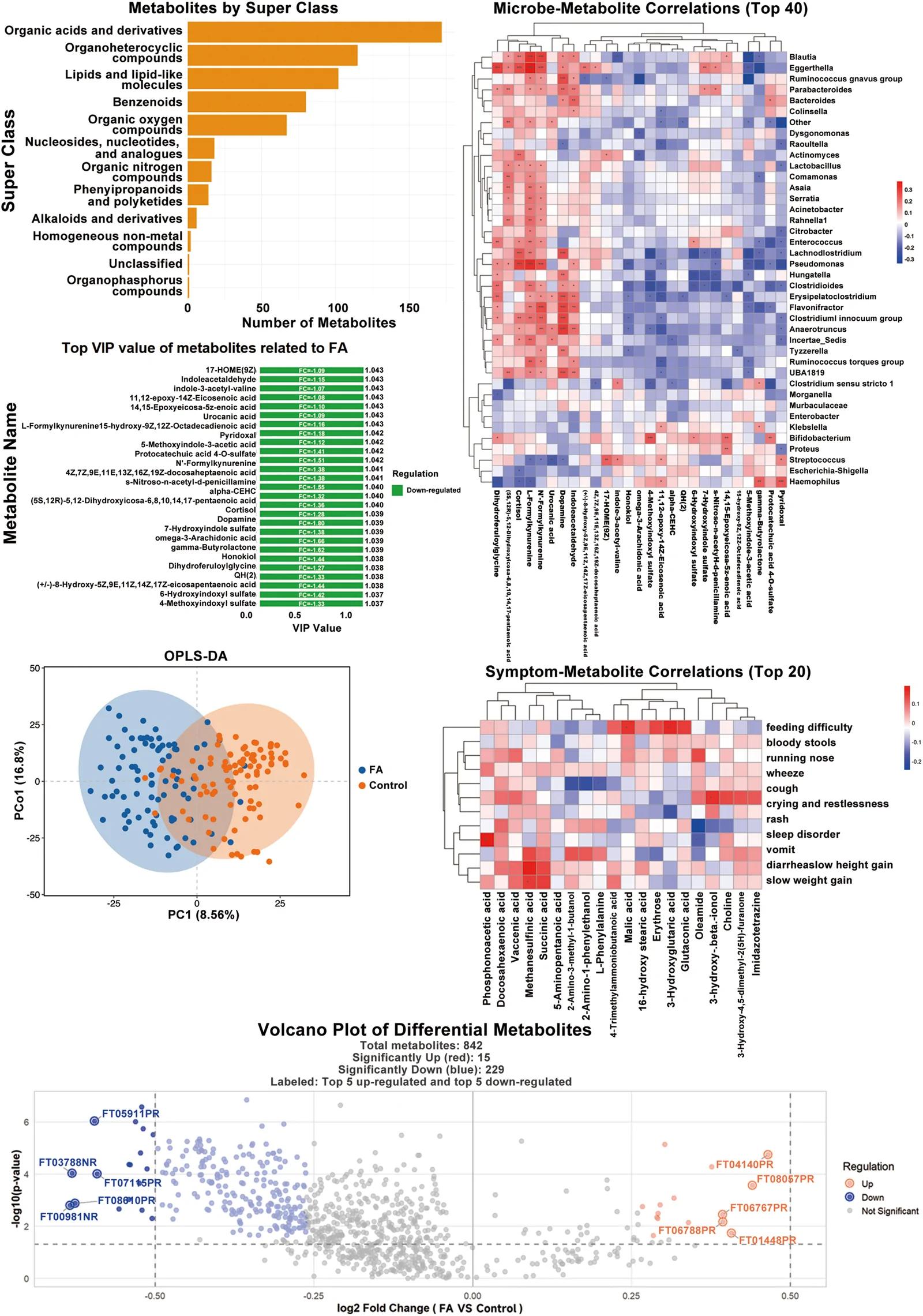
**Metabolism comparisons between FA group (n=87) and Control group (n=90). (A)** Metabolite classification diagram (based on super class). **(B)** The top VIP value of metabolites related to IgE-FA among 3016 metabolites. VIP value is an indicator used to measure the contribution of metabolites to the classification of OPLS-DA model. Metabolites with VIP≥1 are core differential molecules. FC stands for log_2_(Fold Change), indicating the relative expression level of metabolites in the FA group compared to the Control group. Negative log_2_(Fold Change) value indicates downward regulation. **(C)** Heatmap of correlations between microbial genera and metabolites (only display the top 40 items with the highest relevance). The color gradient represents the Spearman correlation coefficient (*r*), with red indicating positive correlations and blue indicating negative correlations. The intensity of color reflects the magnitude of the correlation (range: 0.3 to 0.3). Hierarchical clustering (Euclidean distance, Ward's method) was applied to both microbial genera (y-axis) and metabolites (x-axis) to group entities with similar correlation patterns. ∗ indicates P < 0.05, ∗∗ indicates P < 0.01, ∗∗∗ indicates P < 0.001. **(D)** OPLS-DA two-dimensional score plot of FA group (blue dots) and Control group (orange dots). The horizontal axis represents PC1, and the vertical axis represents PCo1. The 95% confidence ellipses are superimposed to visualize the group distribution tendency. **(E)** The volcano plot of differential metabolites based on 842 significant metabolites in FA group and Control group. Light red dot indicates upregulated metabolites (P < 0.05 and FC > 1.2), light blue dot indicates down-regulated metabolites (P < 0.05 and FC < −1.2), dark blue dot indicates significantly down-regulated metabolites (P < 0.05 and FC < −1.5), gray dot indicates non-significant metabolites. Top 5 up/down-regulated metabolites are labeled in metabolite ID. **(F)** Heat map of correlation analysis between clinical symptoms and metabolites (Top 20). ∗ indicates P < 0.05, ∗∗ indicates P < 0.01.

Following random forest feature selection, 842 significant metabolites were identified. Fig. 3D revealed FA group and Control group exhibited a partially overlapping but distinct metabolic distribution pattern, indicating there were measurable metabolic differences between the FA infants and healthy controls although the separation was not completely distinct. The volcano plot showed that metabolites in the FA group mainly exhibited a downregulated trend, while the upregulated trend was not statistically significant, indicating that cephalosporins primarily exert an inhibitory effect on the gut microbiota and its metabolites (Fig. 3E).

Fig. 3F demonstrates the relationship between top 20 metabolites and clinical symptoms. However, after adjusted P-value correction, only sleep disturbance showed a significant negative correlation with Oleamide, which is the first discovered “sleep lipid' that can induce physiological sleep.^22^

### Enrichment analysis of KEGG pathways and reactome

To further elucidate the functional implications of the altered metabolites, we mapped them to Kyoto Encyclopedia of Genes and Genomes (KEGG) pathways and identified the 20 most frequently enriched pathways (Fig. 4A). Fig. 4B demonstrates the top 10 down-regulated KEGG pathways in FA group. The tryptophan metabolism pathway ranked first, which is consistent with the findings in metabolite analysis. This pathway regulates immunity and oxidative stress. Its downregulation impairs the microbial indole pathway and aryl hydrocarbon receptor (AHR) activation. Simultaneously, increased gut barrier permeability promotes food allergen translocation and a T-helper 2 (Th2) -skewed immune response.

**Fig. 4:**
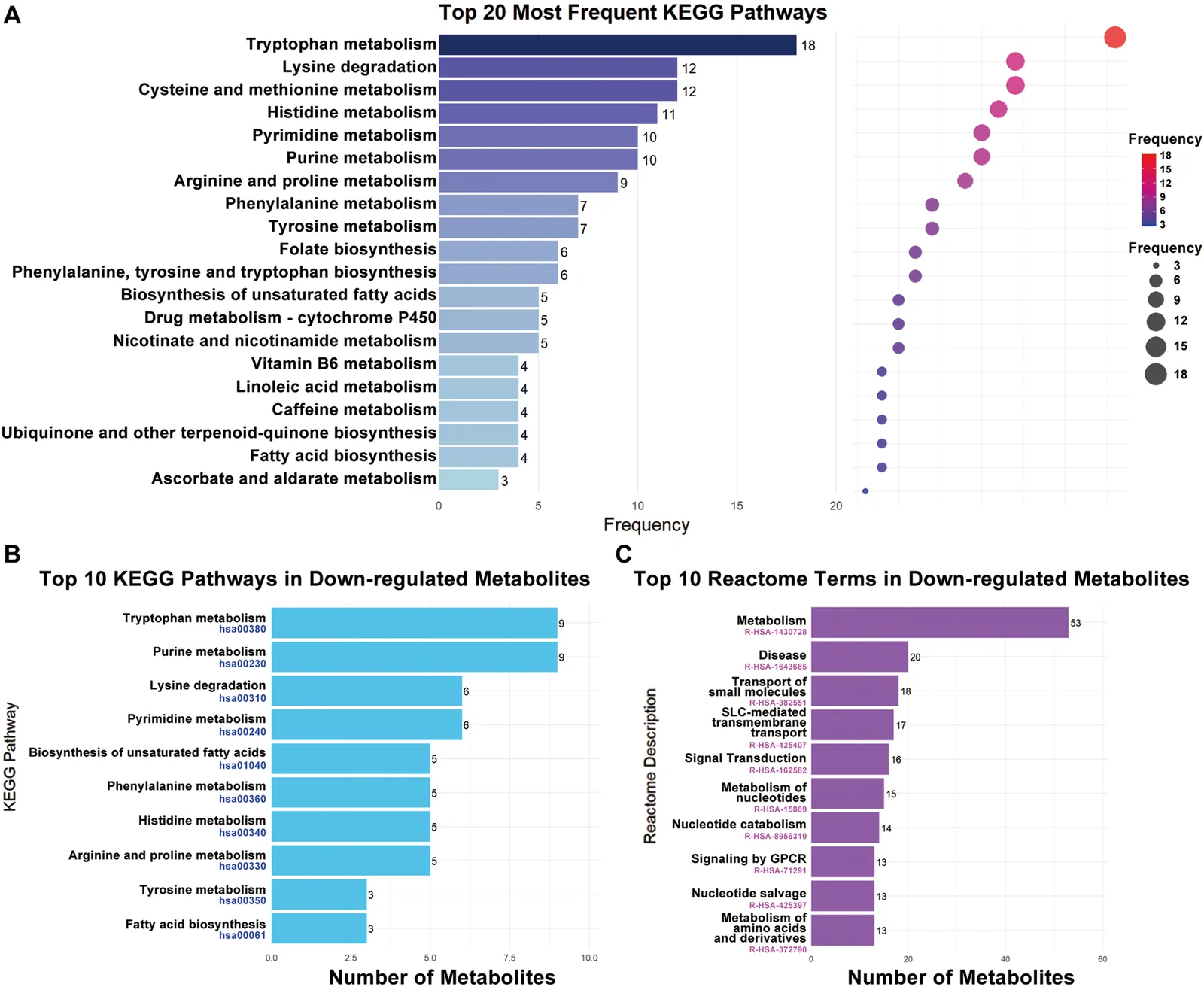
**KEGG pathways and Reactome items comparisons between FA group (n=87) and Control group (n=90). (A)** Top 20 Most Frequent KEGG Pathways in all samples. The y-axis corresponds to the KEGG pathway names and the x-axis represents the enrichment ratio of specific pathways. The dots in the bubble plot indicate the frequency of enrichment. **(B)** Top 10 KEGG pathways enriched in down-regulated metabolites, with the x-axis representing the number of metabolites mapped to each pathway. **(C)** Top 10 Reactome items enriched in down-regulated metabolites, with the x-axis indicating the count of metabolites associated with each Reactome ID.

A significant downregulation was also observed in the arginine metabolism pathway. Arginine metabolism produces anti-inflammatory nitric oxide (NO) and polyamines, which regulate vascular permeability and immune cell proliferation. In addition to the endogenous pathway, a downregulation of an exogenous NO donor, s-Nitroso-n-acetyl-d-penicillamine (SNAP), was also observed in metabolite analysis. Two downregulations of NO generation pathway diminish the anti-inflammatory and vasodilatory effects of NO, potentially exacerbating vascular leakage and tissue edema during allergic reactions, and may also impair the proper functional regulation of immune cells.

Fig. 4C illustrates the top 10 suppressed Reactome terms in the FA group. These down-regulated amino acid metabolic pathways are associated with various diseases, including neurological and immune disorders, particularly the tryptophan metabolism pathway. The down-regulated nucleic acid metabolism pathways are closely linked to cell proliferation and energy metabolism. Lipid metabolism is associated with inflammation, cell membrane function, and energy storage. These findings explain the growth retardation and the fragility of the intestinal and skin barriers in FA children.

## Discussion

Our study specifically focused on cephalosporin antibiotic with precisely defined exposure windows. Furthermore, we controlled for confounders to examine how cephalosporins exposure in early life alters gut microbiota and metabolites, offering micro-level insight into its potential role in the development and clinical manifestations of FA.

Regarding the role of cephalosporins in FA, Hirsch et al^23^ reported that the association between cephalosporins and FA was observed only in children diagnosed before 2 years of age and was not related to milk allergy. However, their data were derived from electronic medical record systems without follow-up study. Our study addressed this limitation and further identified the critical time window of this association. Our study identifies the intrapartum period and the first 2 postnatal months as a critical window for cephalosporins exposure-induced IgE-FA. The effect manifests as an “all-or-none” phenomenon, demonstrating no association with the dose, course, or total frequency of antibiotic exposure, but exhibits a strong synergistic effect with positive family allergic history. We thus propose this pattern functions as a “switch” for FA susceptibility, determining whether genetically predisposed individuals transition to an allergic phenotype. However, due to sample size constraints, subgroup analyses stratified by specific allergens were not performed.

Previous studies have largely attributed the association between antibiotics and FA to gut microbiota dysbiosis. This finding provides a novel paradigm for gene-environment interactions: genetic susceptibility confers the “allergic potential”, while cephalosporins serve as the key environmental trigger activating that potential. Therefore, it is important to weigh the pros and cons of cephalosporins use in infants aged 0–2 months, especially in the presence of a positive family allergic history. Adopting non antibiotic therapy such as microecological therapy or traditional Chinese medicine as much as possible for infants to reduce the use of antibiotics is recommended. As for intrapartum exposure, although the OR is only 1.04, given the widespread use of antibiotics in obstetrics, even a weak association may have significant public health implications. In alignment with obstetric antibiotic guidelines,^13^ we recommend that fetal delivery should ideally occur within 0.25–6 h following prophylactic antibiotic administration.

Our analysis confirmed significant dysbiosis in cephalosporins exposure-induced IgE-FA infants, marked by enrichment of pro-inflammatory genera (eg, *Escherichia_Shigella*) and depletion of beneficial bacteria (eg, *Bifidobacterium*, *Lactobacillus*).^24^, ^25^, ^26^, ^27^ These shifts may promote Th2-skewed immunity and compromise intestinal barrier integrity.^28^, ^29^, ^30^ So we hypothesized early-life cephalosporins exposure disrupts neonatal gut microbiota colonization during a critical window for immune development,^31^ thereby increasing FA risk.^19^ Based on our findings, supplementing infants with potentially depleted beneficial bacteria after early cephalosporin exposure may mitigate FA risk. Furthermore, our symptom–microbiota association map may inform novel strategies for symptom management, whereas our microbiota-based FA prediction model establishes a basis for the future development of non-invasive diagnostic tools.

Metabolomic and pathway analyses further revealed disruptions in tryptophan metabolism and NO biosynthesis, alongside downregulation of anti-inflammatory pathways. Adriaan et al demonstrated that tryptophan is essential for maintaining immune tolerance and gut microbiota abundance.^32^ Impaired tryptophan metabolism may reduce AHR activation,^33^ a key regulator of mucosal immunity, while diminished NO production which may exacerbate allergic inflammation. These alterations likely underlie key pathological mechanisms of FA: impaired barrier function may promote allergen translocation, while weakened immunoregulatory signals could favor a pro-allergic Th2 response. Our study bridges specific metabolic perturbations with systemic immune dysregulation, providing a novel, metabolite-centric framework for understanding FA pathogenesis.

Previous studies have largely investigated genetic or environmental factors for FA in isolation, without elucidating their potential interaction or the molecular mechanisms involved. Our findings refined the cephalosporins exposure time window and carried important clinical implications for rational antibiotic prescribing in obstetric care and young infants, especially those with a family history of atopy. The identified disturbances in gut microbiota composition and metabolic pathways provide new insights into gene-environment interactions and open new avenues for non-invasive IgE-FA diagnosis. Moreover, the findings of this study provide clues for potential preventive and therapeutic targets, such as probiotic administration and metabolic modulation, although these remain exploratory and require further validation before clinical translation.

### Limitations

This study has certain limitations. First, due to most of the pregnant women in our hospital are residents of southern coastal region and parents with positive family allergic history are more willing to enroll, the reported allergy rate can not reflect the regional rate, and extrapolation should be cautious. Second, allergy symptoms and suspected foods were first reported by the parents, a potential for reporting bias cannot be excluded. Third, findings related to specific metabolites and pathways remain preliminary and require further mechanistic validation to establish causality. Fourth, although the total sample size reached a certain scale, the relatively small sample size in analyses (mainly cephalosporins exposure within 0–1 months of age) for specific exposure factors resulted in wide confidence intervals. Future cohort studies targeting specific exposure factors are needed to further prove our findings.

## Conclusion

Our study identifies early-life cephalosporins exposure as a risk factor for IgE-FA, which may function by acting as a “switch' and restructuring gut microbial communities. These findings underscore the importance of precise administration of obstetric antibiotics and optimization of early-life antibiotic use. Furthermore, the identified correlations between specific gut microbiota/metabolites and FA may contribute to a better understanding of the underlying mechanisms and provide new directions for future research.

## Data availability

The raw sequencing and metabolomic data generated in this study have been deposited in relevant international repositories. Specifically, 16 S rRNA gene sequencing data from the human cohorts areavailable under NCBI BioProject accession numbers PRJNA1279265. Transcriptomic sequencing data have been submitted to the NCBI GEO database under accession number GSE300252. The datasets containing maternal and infant privacy information are available from the corresponding author on reasonable request.

## Ethics approval

This study was performed in line with the principles of the Declaration of Helsinki. Approval was granted by the Ethics Committee of The Second Affiliated Hospital and Yuying Children's Hospital of Wenzhou Medical University, Wenzhou (2022-K-339-01). All participants were informed of the research objectives, methods and objectives.

## Funder statement

This study was funded by Wenzhou Science and Technology Bureau, Zhejiang Provincial Department of Science and Technology and Zhejiang Provincial Clinical Research Center. All funders had no role in the conduct of the research.

## Authorship contributions

Ning Zhu: Conceptualization (lead); writing – original draft (lead); formal analysis (lead); writing – review and editing (equal). Yimin Zhao: Investigation (lead); writing – review and editing (equal). Fangyuan Cai: Software (lead); visualization (lead). Huixi Tu: Writing – review and editing (equal). Dr Zhangye Xu: Resources (lead); conceptualization (supporting); Writing – review and editing (supporting). Yuexin Gu: Data curation (lead). Xiaoye Lv: Data Curation (equal). Dr Lei Wang: Methodology (lead); writing – original draft (equal). Dr Weixi Zhang: Conceptualization (equal); funding acquisition (lead); project administration (lead). All authors approved the final manuscript as submitted and agree to be accountable for all aspects of the work.

## Disclosure statement of artificial intelligence (AI)

Nothing to disclose.

## Funding

This work was supported by the Major Project of 10.13039/501100007194Wenzhou Science and Technology Bureau (No. ZY2022011) and the "Pioneering Leadership+X" Research and Development Plan of 10.13039/501100008990Zhejiang Provincial Department of Science and Technology (No. 2024C03237), as well as Zhejiang Provincial Clinical Research Center for Pediatric Precision Medicine.

## Disclosure of conflicts of interest

The authors declare that they have no conflicts of interest.
