# Supplementary material for: Prospective birth cohort: Cephalosporins drive gut microbiota and food allergy
Source: World Allergy Organ J. 2026 Oct 8;19(10):101475. doi: 10.1016/j.waojou.2026.101475 (PMC13675632; doi:10.1016/j.waojou.2026.101475)
Supplement: Supplementary file 2 [file mmc2.docx]

**Version date: January 21, 2024**

**Informed Consent Form Notification Page (Translated version)**

**Informed Consent Form (Legal Guardian Version)**

**Experiment name:** Construction and Application of Precise Diagnosis Model for Children's Food Allergy Based on Medical Big Model and Multi omics

**Research units:** The Second Affiliated Hospital and Yuying Children's Hospital of Wenzhou Medical University, Wenzhou, 325027, China.

We invite you to participate in an experiment. Before deciding to participate in this trial, please read this informed consent form carefully. If you have any questions that you do not understand, you may request an explanation of any terms or information that you are unclear about from the researcher or members of the trial working group responsible for the trial.

1. **Research background and purpose**

**1.1 The situation of preventing and controlling food allergies in children is severe, and early diagnosis is a difficult problem**

Food allergy (FA) is an abnormal response of the immune system to certain food allergens, with a global prevalence of 6% -8% in children, making it the third most common disease among children under 18 years old. In addition to the increasing incidence of illness, the severity of food allergies in children is also increasing. About 40% of affected children may experience severe allergic reactions, even leading to death. In the past decade, the incidence of food allergies in children in China has shown a significant upward trend, and the number of hospitalizations due to food allergies has also increased. This is the "second wave of allergy epidemics" after the rising incidence of bronchial asthma (asthma). Taking Chongqing as an example, the incidence of food allergies in children under 2 years old has increased by 120% and 217% in the past 10 and 20 years, respectively, and reached 11.1% in 2019. At present, there are over 20 million children with food allergies in China, and the prevention and control situation is extremely severe: food patients can experience various symptoms ranging from mild to severe, affecting the digestive system, skin system, respiratory system, etc. In extreme cases, it can cause serious allergic reactions including anaphylactic shock, often endangering lives and causing heavy blows to families. Even if timely treatment is received, it can bring psychological pressure to the children and affect their normal life. However, currently, for children with food allergies, hospitals in various regions do not have a strict grasp of the diagnosis and treatment standards for food allergies, and the level of diagnosis and treatment varies greatly. They are unable to make timely and accurate diagnoses, which affects the timely treatment of patients. This not only damages the quality of life and psychological health of children and their families, but also may affect the growth and development of children. The increase in the number of food allergy patients and the deepening public attention to their own food allergies have promoted the development of the food allergy industry. This means that the demand for early diagnosis and management of food allergies is also increasing. It is estimated that the annual consumption scale of the national allergy market exceeds 20 billion yuan, and the total scale of the children's allergy consumption market exceeds 50 billion yuan, which provides broad development prospects for the food allergy industry. Compared to this, there are only over 100 hospitals in China that have established departments related to childhood allergies, resulting in insufficient diagnostic and treatment capabilities for childhood allergies and a lack of resource investment. Therefore, it is particularly important to focus on the prominent issue of allergic diseases in children, accelerate the establishment of early warning screening and evaluation systems, and develop new diagnostic strategies.

**1.2 Research Objective**

This project aims to utilize a medical language model optimized based on medical papers to achieve accurate diagnosis of food allergies in children without the need for trigger tests, thereby improving patient safety. We plan to integrate clinical data from a multicenter prospective cohort study and multi omics analysis of children's food allergies, in order to add professional domain knowledge, further improve the diagnostic accuracy and reasoning ability of the model, achieve accurate diagnosis of children's allergies, and predict targets and high-risk factors, thereby reducing medical misdiagnosis, diagnosis, and labor costs.

2. **Research Methods**

**2.1 Who can participate in the experiment?**

① Both parents have resided in Wenzhou for more than 2 years;

② Both parents have no history of infectious diseases such as HIV and other sexually transmitted diseases, and no family hereditary diseases such as Down syndrome;

③ Single pregnancy and no maternal complications (pregnancy diabetes, hypertension and eclampsia);

④ Both parents agree to participate and are willing to cooperate with the follow-up until the child is 2 years old (signing an informed consent form).

**2.2 Who cannot participate in the experiment?**

① Lack of baseline clinical data and questionnaire surveys;

② The infant suffers from serious congenital diseases (congenital heart disease, bronchopulmonary dysplasia, biliary atresia, diabetes, etc.), or is found to suffer from potentially serious diseases such as tumors during follow-up;

③ Infants and young children have a history of mechanical ventilation.

④ Individuals who experience intolerable adverse events, serious adverse events, or clinically significant laboratory abnormalities.

**2.3 Research and follow-up methods**

**2.3.1 Research subjects and groups**

Establish a birth cohort based on the inclusion criteria and divide them into a food allergy group and a non food allergy group during follow-up until one year old. A total of 2000 mother infant pairs will be included, of which our center will undertake 800 pairs.

**2.3.2 Follow up time**

Follow up with the mother during pregnancy, at birth, at 6 months and 12 months of age to determine the food allergy group and non food allergy group. Among them, parents need to fill out relevant survey questionnaires at the time of enrollment, when the child is 6 months old, and when the child is 12 months old. They need to cooperate in sending out a stool sample when the child is 6 months old, and undergo blood allergen testing at the hospital designated by the researcher when the child is 12 months old.

**3. Research risks and benefits, costs and compensation**

**3.1 What risks will my child encounter in the research?**

Your child will not have any additional risks in the study.

**3.2 What are the possible benefits for my child?**

For children with food allergies, this project is a new exploration of precise diagnosis of children's food allergies, and is expected to become an intelligent new tool for children's allergy research, providing new ideas for the precise diagnosis and target and high-risk factor prediction system of children's allergies in China.

After joining the group, you will receive a one-year free online consultation, free testing of gut microbiota at 6 months of age, and free testing of blood allergens at 12 months of age.

**3.3 Do I need to pay to participate in this study?**

You do not need to pay, the cost of collecting and shipping feces and the cost of blood allergen testing will be borne by the researcher.

**3.4 What compensation can I receive for participating in this study?**

We will not compensate you for participating in this study.

**4. The rights you possess**

You have the right to decide whether to participate in this experiment. If you cannot make a decision immediately, you have sufficient time to consider it. If necessary, you can consult with relatives, friends, and other trusted individuals before making a decision.

If you decide not to participate in this experiment, it will not affect your relationship with the researcher, you will not face discrimination or retaliation, and your treatment and rights will not be affected.

If you decide to participate in this trial, we hope you can complete the trial without special reasons, but you have the right to withdraw at any time during the trial period. If you decide to withdraw, please inform the researcher promptly.

During the trial period, you can always access information and materials related to you in this trial.

**5. Privacy Protection**

The personal information you provide to the researchers (such as name, gender, contact information, survey questionnaire, etc.) may also be obtained by the following personnel in addition to normal research needs: staff of the research funding institution related to this experiment (supervisors, inspectors, etc.)

But no one is allowed to disclose your personal information to others or other institutions without your permission. Except for researchers and administrative agencies, no other person or organization has the right to actively contact you about this experiment or directly provide you with information about this experiment.

The results of this experiment may be published in academic papers, but your child's personal information will not appear in any publicly available documents.

6. **Other**

**6.1 When the following situations occur, for the sake of your health, the researcher may withdraw you from this trial without your consent:**

Upon joining the group, it was found that the candidate did not meet the selection criteria or met any of the exclusion criteria;

Continuing to participate in this trial may result in your risks outweighing your benefits;

When a disease with potential serious adverse outcomes (tumor) is discovered during follow-up;

Serious violation of the plan;

Other researchers deemed it inappropriate to continue participating in this study and terminated the trial prematurely.

**6.2 This informed consent form is in duplicate, with the researcher and you each keeping one copy**

**7. Compensation for injuries incurred during the experiment**

If your injury is directly caused by participating in this trial, you do not have to pay any medical expenses for treatment, which will be borne by the researcher.

**8. Contact Information**

During your participation in this clinical study, if you have any questions or urgent situations, please contact your research doctor at 13857724836.

Inform the subjects that the research doctor will provide you with detailed explanations to fully understand the above content and give you sufficient time to consider and make a decision on whether to participate in the study.

During the research period, if any information that may affect the subjects' continued participation in the trial is obtained, the subjects or their legal representatives will be notified in a timely manner. If necessary, a newly signed informed consent form will be obtained from you.

This experimental protocol has been approved by the Ethics Committee of the Second Affiliated Hospital and Yuying Children's Hospital of Wenzhou Medical University. If you have any dissatisfaction during the study, please contact the Medical Ethics Committee of the Second Affiliated Hospital and Yuying Children's Hospital of Wenzhou Medical University.

Ethics Committee Office: Ethics Committee Office of Longwan Branch of The Second Affiliated Hospital and Yuying Children's Hospital of Wenzhou Medical University.

Contact person: Teacher Chen Contact phone number: 0577-85676879

Thank you for reading the above materials. If you decide to participate in this experimental study, please inform your doctor and he/she will arrange all matters related to the trial for you.

Please keep this information.

**Informed Consent Form (Signature Page)**

1. I have carefully read the instructions for the subjects and understood the relevant background of this trial. The researcher has provided me with a detailed explanation of the characteristics of the study and possible adverse reactions, and answered my questions

2. I know that if I refuse to participate in this trial, my treatment and rights will not be affected. After understanding all the information about the subjects and fully considering it, I voluntarily participate in this trial

3. I am willing to follow the instructions of the researcher and participate in the experiment according to the research protocol. During the experiment, I have the right to withdraw at any time, but before withdrawing, I need to inform the researcher in a timely manner.

During the trial period, if any discomfort symptoms occur, I will promptly inform the researchers.

**Subject signature:**

**Signature of the representative/guardian of the subject:**

**Signature Date:**

**Relationship between Agent/Guardian and Subject:**

**Signature of researcher:**

**Signature Date:**

**Version date: November 29, 2022**

**Informed Consent Form Notification Page (Translated version)**

**Informed Consent Form (Legal Guardian Version)**

**Experiment name:** Exploring the protective effect and mechanism of oligofructose on food allergies after exposure to cephalosporin antibiotics based on the "Allergic Disease Cohort" - single center, double-blind, and randomized controlled study.

**Research units:** The Second Affiliated Hospital and Yuying Children's Hospital of Wenzhou Medical University, Wenzhou, 325027, China.

We invite you to participate in an experiment. Before deciding to participate in this trial, please read this informed consent form carefully. If you have any questions that you do not understand, you may request an explanation of any terms or information that you are unclear about from the researcher or members of the trial working group responsible for the trial.

**1. Research background and purpose**

**1.1 Research background**

Food allergy in children is the third most common disease among children under 18 years old, with a prevalence rate of up to 6% to 8% globally. In recent years, it has shown a significant upward trend in China. Taking Chongqing as an example, the number of children under 2 years old has increased by 120% and 217% respectively in the past 10 and 20 years, reaching as high as 11.1% in 2019. The incidence of food allergies among children in some regions can even reach 21.13%, significantly higher than the international level. In addition to the increasing incidence of illness, the severity of food allergies in children is also increasing. Our team found that food allergies in children aged 3-6 mainly manifest as mild clinical symptoms such as rash, itching, lip pain, abdominal pain, diarrhea, and vomiting. However, about 40% of children may experience severe allergic reactions, even anaphylactic shock, which can lead to death. Moreover, food allergies often progress to other allergic diseases such as bronchial asthma (abbreviated as asthma) and allergic rhinitis during the pre-school and school years. Due to the fact that food allergies are often incurable, avoiding palliative treatment for allergens can lead to complications such as malnutrition and developmental delay, even if symptoms are alleviated, becoming another major clinical problem for allergic diseases after asthma.

Fructooligosaccharides (FOS) are a common prebiotic that regulate the balance of gut microbiota, affect the metabolic pathways of gut microbiota, and have various immunomodulatory effects through fermentation by gut microbiota. The preliminary research of the research group found that FOS can improve food allergy symptoms in mice. So, can FOS alleviate food allergy symptoms induced by exposure to cephalosporin antibiotics? It has not been reported yet. This study will conduct a prospective randomized controlled trial of FOS in the treatment of food allergies based on a prospective food allergy cohort, to clarify whether FOS can improve food allergy symptoms after exposure to cephalosporin antibiotics, and to deeply investigate its potential mechanisms, providing new strategies for effectively reversing food allergies in infants and young children exposed to cephalosporin antibiotics in early life.

**1.2 Research objective**

The association between the use of cephalosporin antibiotics in early life and food allergies in children, and further clarification of the protective effect and related mechanisms of oligofructose on food allergies after exposure to cephalosporin antibiotics, has important social significance and potential social value

**2. Research Methods**

1. Who can participate in the experiment?

① Both parents have resided in Wenzhou for more than 2 years;

② Both parents have no history of infectious diseases such as HIV and other sexually transmitted diseases, and no family hereditary diseases such as Down syndrome;

③ Single pregnancy and no maternal complications (pregnancy diabetes, hypertension and eclampsia);

④ Both parents agree to participate and are willing to cooperate with the follow-up until the child is 2 years old (signing an informed consent form).

2. Who cannot participate in the experiment?

① Lack of baseline clinical data and questionnaire surveys;

② The infant suffers from serious congenital diseases (congenital heart disease, bronchopulmonary dysplasia, biliary atresia, diabetes, etc.), or is found to suffer from potentially serious diseases such as tumors during follow-up;

③ Infants and young children have a history of mechanical ventilation.

④ Individuals who experience intolerable adverse events, serious adverse events, or clinically significant laboratory abnormalities.

**3. Research methods and statistical processing**

**3.1 Research subjects and groups**

It will take approximately 2 years for your child to participate in and complete this study. There will be 800 patients enrolled in this study. Establish a birth cohort based on the inclusion criteria and divide them into a food allergy group and a non food allergy group during follow-up until one year old. One year old children with cephalosporin antibiotic related food allergies were selected as the second layer of research subjects, and were randomly divided into a FOS intervention group and a control group in a 1:1 ratio.

Oligofructose intervention group: Oral administration of oligofructose solution, once daily, 10ml, for 4 weeks, during which the child is still required to undergo food avoidance treatment. Control group: Oral administration of 5% glucose solution, once daily, 10ml, for 4 weeks, during which the child is still required to undergo food avoidance treatment.

**3.2 Follow up time**

Follow up with the mother during pregnancy, at birth, at 6 months and 12 months of age to determine the food allergy group and non food allergy group. Further study will focus on 1-year-old children with cephalosporin antibiotic related food allergies as the second tier research subjects, lasting for 4 weeks.

**3.3 Clinical efficacy evaluation**

① Main evaluation indicators: changes in the severity of food allergies and changes in the proportion of children with improved, unchanged, or worsened clinical manifestations.

② Secondary evaluation indicators: Secondary evaluation indicators: Extract medical records and record the growth and development (height and weight), anemia, hypoalbuminemia, and incidence and frequency of other allergic diseases (atopic dermatitis, allergic rhinitis) within 6 and 12 months after the intervention of the child. Detect allergen specific biomarker levels, such as specific IgE serum concentration, eosinophils.

**4. Research risks and benefits, costs and compensation**

**4.1 What risks will I encounter in my research?**

The treatment you received in the study was oral administration of oligofructose solution, which is a common prebiotic and does not increase additional risks. If any discomfort occurs during this period, such as coughing, bloating, nausea, vomiting, rash, or other adverse reactions, please inform the doctor in a timely manner, and the doctor will take corresponding measures according to your situation. 2. What are the possible benefits I may gain

For children with food allergies, this study may reverse or improve food allergy reactions, but it may not be helpful. However, we hope that the information obtained from this project will help clarify the association and underlying mechanisms between the use of cephalosporin antibiotics in early life and food allergies in children.

**4.2 Do I need to pay to participate in this study?**

The treatment you may receive in your research, which is oral oligofructose solution, does not require payment, and the collection of various information on you is also based on the information generated during your normal life or when seeking medical treatment for illness.

**4.3 What compensation can I receive for participating in this study?**

We will not compensate you for participating in this study.

5. **The rights you possess**

You have the right to decide whether to participate in this experiment. If you cannot make a decision immediately, you have sufficient time to consider it. If necessary, you can consult with relatives, friends, and other trusted individuals before making a decision.

If you decide not to participate in this experiment, it will not affect your relationship with the researcher, you will not face discrimination or retaliation, and your treatment and rights will not be affected.

If you decide to participate in this trial, we hope you can complete the trial without special reasons, but you have the right to withdraw at any time during the trial period. If you decide to withdraw, please inform the researcher promptly.

During the trial period, you can always access information and materials related to you in this trial.

**6. Privacy Protection**

The personal information you provide to the researchers (such as name, gender, contact information, survey questionnaire, etc.) may also be obtained by the following personnel in addition to normal research needs: staff of the research funding institution related to this experiment (supervisors, inspectors, etc.).

But no one is allowed to disclose your personal information to others or other institutions without your permission. Except for researchers and administrative agencies, no other person or organization has the right to actively contact you about this experiment or directly provide you with information about this experiment.

The results of this experiment may be published in academic papers, but your personal information will not appear in any publicly available documents.

**7. Other**

**7.1 When the following situations occur, for the sake of your health, the researcher may withdraw you from this trial without your consent:**

Upon joining the group, it was found that the candidate did not meet the selection criteria or met any of the exclusion criteria;

Continuing to participate in this trial may result in your risks outweighing your benefits;

When a disease with potential serious adverse outcomes (tumor) is discovered during follow-up;

Serious violation of the plan;

Other researchers deemed it inappropriate to continue participating in this study and terminated the trial prematurely.

**7.2 This informed consent form is in duplicate, with the researcher and you each keeping one copy**

**7.3 Compensation for injuries incurred during the experiment**

If your injury is directly caused by participating in this trial, you do not have to pay any medical expenses for treatment, which will be borne by the researcher.

**8. Contact Information**

During your participation in this clinical study, if you have any questions or urgent situations, please contact your research doctor at 13857724836.

Inform the subjects that the research doctor will provide you with detailed explanations to fully understand the above content and give you sufficient time to consider and make a decision on whether to participate in the study.

During the research period, if any information that may affect the subjects' continued participation in the trial is obtained, the subjects or their legal representatives will be notified in a timely manner. If necessary, a newly signed informed consent form will be obtained from you. This experimental protocol has been approved by the Ethics Committee of the Second Affiliated Hospital and Yuying Children's Hospital of Wenzhou Medical University. If you have any dissatisfaction during the study, please contact the Medical Ethics Committee of the Second Affiliated Hospital and Yuying Children's Hospital of Wenzhou Medical University at 0577-88002560, or email feikikcy@ 126.com

**Informed Consent Form (Signature Page)**

1. I have carefully read the instructions for the subjects and understood the relevant background of this trial. The researcher has provided me with a detailed explanation of the characteristics of the study and possible adverse reactions, and answered my questions

2. I know that if I refuse to participate in this trial, my treatment and rights will not be affected. After understanding all the information about the subjects and fully considering it, I voluntarily participate in this trial

3. I am willing to follow the instructions of the researcher and participate in the experiment according to the research protocol. During the experiment, I have the right to withdraw at any time, but before withdrawing, I need to inform the researcher in a timely manner.

During the trial period, if any discomfort symptoms occur, I will promptly inform the researchers.

**Subject signature:**

**Signature of the representative/guardian of the subject:**

**Signature Date:**

**Relationship between Agent/Guardian and Subject:**

**Signature of researcher:**

**Signature Date:**
