# Supplementary material for: Prospective birth cohort: Cephalosporins drive gut microbiota and food allergy
Source: World Allergy Organ J. 2026 Oct 8;19(10):101475. doi: 10.1016/j.waojou.2026.101475 (PMC13675632; doi:10.1016/j.waojou.2026.101475)
Supplement: Supplementary file 3 [file mmc3.docx]

**Appendices**

**Table S1.** Combined analysis of family allergic history and the time of intrapartum cephalosporins exposure.

|  | Number of cases | P-value | OR |
| --- | --- | --- | --- |
| Independent stratified analysis | | | |
| 0-6 hours before fetal expulsion | 351 | 0.938 | 0.988 |
| 6-12 hours before fetal expulsion | 44 | 0.523 | 1.249 |
| 12-18 hours before fetal expulsion | 21 | 0.303 | 1.615 |
| 18-24 hours before fetal expulsion | 18 | 0.587 | 1.338 |
| Combined with family allergic history (n = 335) | | | |
| 0-6 hours before fetal expulsion | 123 | 0.053 | 1.514 |
| 6-12 hours before fetal expulsion | 16 | 0.110 | 2.347 |
| 12-18 hours before fetal expulsion | 7 | 0.044 | 4.693 |
| 18-24 hours before fetal expulsion | 4 | 0.112 | 7.040 |

**Table S2.** Table of confounding factors in intestinal flora analysis (FA group : Control group = 129 : 192).

|  | Level | Control group | FA group | Statistic | P-value |
| --- | --- | --- | --- | --- | --- |
| Birth characteristic | | | | | |
| gender | male | 85 | 70 | -1.754 | 0.079 |
|  | female | 107 | 59 |  |  |
| delivery mode | vaginal delivery | 149 | 101 | -0.146 | 0.884 |
|  | Cesarean section | 43 | 28 |  |  |
| birth weight (average, g) | | 3236.8 | 3257.5 | -0.353 | 0.724 |
| fetal gestational age (average, week) | | 38.7 | 38.9 | -1.743 | 0.081 |
| family allergic history | no | 121 | 75 | -0.878 | 0.380 |
|  | yes | 71 | 54 |  |  |
| Feeding characteristic | | | | | |
| feeding practice | breastfeeding primarily | 178 | 118 | 0.876 | 0.645 |
|  | mixed feeding | 3 | 1 |  |  |
|  | formula feeding primarily | 11 | 10 |  |  |
| age for introducing complementary food | 4 months of age | 1 | 1 | 2.256 | 0.689 |
|  | 5 months of age | 54 | 36 |  |  |
|  | 6 months of age | 82 | 57 |  |  |
|  | no | 55 | 35 |  |  |
| complementary food in the past 1 month | |  |  |  |  |
| rice or noodles | almost every day | 28 | 26 | 0.089 | 0.993 |
|  | 1-3 times per week | 26 | 16 |  |  |
|  | 1-3 times per month | 6 | 4 |  |  |
|  | none | 122 | 83 |  |  |
| corn, chestnut rice, millet | almost every day | 3 | 2 | 0.120 | 0.989 |
|  | 1-3 times per week | 4 | 2 |  |  |
|  | 1-3 times per month | 3 | 2 |  |  |
|  | none | 182 | 123 |  |  |
| vegetables | almost every day | 10 | 7 | 3.029 | 0.387 |
|  | 1-3 times per week | 26 | 18 |  |  |
|  | 1-3 times per month | 0 | 2 |  |  |
|  | none | 156 | 102 |  |  |
| fruits | almost every day | 5 | 4 | 1.893 | 0.755 |
|  | 1-3 times per week | 23 | 15 |  |  |
|  | 1-3 times per month | 5 | 7 |  |  |
|  | none | 159 | 103 |  |  |
| meat | almost every day | 2 | 1 | 0.182 | 0.981 |
|  | 1-3 times per week | 8 | 6 |  |  |
|  | 1-3 times per month | 1 | 1 |  |  |
|  | none | 181 | 121 |  |  |
| liver and other animal viscera | almost every day | 1 | 1 | 4.615 | 0.329 |
|  | 1-3 times per week | 3 | 4 |  |  |
|  | 1-3 times per month | 0 | 2 |  |  |
|  | none | 188 | 122 |  |  |
| fish, shrimp, and crab | almost every day | 1 | 1 | 0.244 | 0.970 |
|  | 1-3 times per week | 1 | 1 |  |  |
|  | 1-3 times per month | 1 | 1 |  |  |
|  | none | 189 | 126 |  |  |
| egg | almost every day | 2 | 2 | 0.560 | 0.906 |
|  | 1-3 times per week | 6 | 5 |  |  |
|  | 1-3 times per month | 3 | 3 |  |  |
|  | none | 181 | 119 |  |  |
| cheese | almost every day | 1 | 1 | 0.750 | 0.687 |
|  | 1-3 times per week | 1 | 0 |  |  |
|  | 1-3 times per month | 0 | 0 |  |  |
|  | none | 190 | 128 |  |  |
| nut | almost every day | 0 | 0 | NA | 1.000 |
|  | 1-3 times per week | 0 | 0 |  |  |
|  | 1-3 times per month | 0 | 0 |  |  |
|  | none | 192 | 129 |  |  |
| soy or legume products | almost every day | 1 | 0 | 0.750 | 0.687 |
|  | 1-3 times per week | 0 | 0 |  |  |
|  | 1-3 times per month | 1 | 1 |  |  |
|  | none | 190 | 128 |  |  |
| iron | none | 178 | 114 | 4.631 | 0.099 |
|  | occasionally | 6 | 11 |  |  |
|  | often | 8 | 4 |  |  |
| zinc | none | 177 | 119 | 1.098 | 0.577 |
|  | occasionally | 9 | 8 |  |  |
|  | often | 6 | 2 |  |  |
| calcium | none | 165 | 111 | 0.070 | 0.966 |
|  | occasionally | 8 | 6 |  |  |
|  | often | 19 | 12 |  |  |
| Vitamin A, D | none | 22 | 14 | 0.045 | 0.978 |
|  | occasionally | 11 | 7 |  |  |
|  | often | 159 | 108 |  |  |
| nitrogen compounds | none | 181 | 119 | 0.545 | 0.761 |
|  | occasionally | 4 | 4 |  |  |
|  | often | 7 | 6 |  |  |
| unsaturated fatty acid | none | 168 | 111 | 2.249 | 0.325 |
|  | occasionally | 8 | 10 |  |  |
|  | often | 16 | 8 |  |  |
| Take probiotics in the past 1 month | no | 189 | 126 | -0.494 | 0.621 |
|  | yes | 3 | 3 |  |  |
| Cephalosporins exposure | | | | | |
| obstetric exposure | no | 132 | 90 | -0.193 | 0.847 |
|  | yes | 60 | 39 |  |  |
| obstetric exposure dose | none | 132 | 90 | -0.131 | 0.896 |
|  | 1 IU | 59 | 37 |  |  |
|  | 1.5 IU | 1 | 2 |  |  |
| obstetric exposure interval (average, h) | | 1.68 | 1.85 | -0.007 | 0.994 |
| 0-7 day of age exposure | no | 185 | 120 | -1.342 | 0.179 |
|  | yes | 7 | 9 |  |  |
| 0-1 months of age exposure | no | 179 | 100 | -4.086 | <0.001 |
|  | yes | 13 | 29 |  |  |
| 1-2 months of age exposure | no | 183 | 119 | -1.139 | 0.255 |
|  | yes | 9 | 10 |  |  |
| 2-6 months of age exposure | no | 168 | 119 | -1.353 | 0.176 |
|  | yes | 24 | 10 |  |  |
| reasons for cephalosporins exposure during 0-6 months of age | fever | 6 | 10 | -1.865 | 0.062 |
|  | cough | 23 | 22 | -1.282 | 0.200 |
|  | vomit | 0 | 1 | -1.220 | 0.222 |
|  | diarrhea | 6 | 9 | -1.601 | 0.109 |
|  | other/unknown | 15 | 15 | -1.150 | 0.250 |

Note: The Mann-Whitney U test was used for binary categorical variables and numeric variable, and the Kruskal-Wallis test was used for multicategorical variables. The data presented in the "Birth characteristic" section are the results of multivariate regression analysis. P>0.05 indicates no statistical difference between the two groups

**Table S3.** Table of confounding factors in metabolites analysis (FA group : Control group = 87 : 90).

|  | Level | | Control group | FA group | Statistic | P-value |
| --- | --- | --- | --- | --- | --- | --- |
| Birth characteristic | | | | | | |
| gender | male | | 39 | 48 | -1.571 | 0.116 |
|  | female | | 51 | 39 |  |  |
| delivery mode | vaginal delivery | | 70 | 71 | -0.631 | 0.528 |
|  | Cesarean section | | 20 | 16 |  |  |
| birth weight (average, g) | | | 3186.0 | 3258.3 | -1.080 | 0.280 |
| fetal gestational age (average, week) | | | 38.7 | 39.1 | -1.840 | 0.066 |
| family allergic history | no | | 56 | 52 | -0.333 | 0.739 |
|  | yes | | 34 | 35 |  |  |
| Feeding characteristic | | | | | | |
| feeding practice | breastfeeding primarily | | 85 | 80 | 1.909 | 0.385 |
|  | mixed feeding | | 1 | 0 |  |  |
|  | formula feeding primarily | | 4 | 7 |  |  |
| age for introducing complementary food | 4 months of age | | 0 | 1 | 4.742 | 0.192 |
|  | 5 months of age | | 26 | 23 |  |  |
|  | 6 months of age | | 32 | 42 |  |  |
|  | no | | 32 | 21 |  |  |
| complementary food in the past 1 month | | |  |  |  |  |
| rice or noodles | almost every day | | 13 | 19 | 6.303 | 0.098 |
|  | 1-3 times per week | | 17 | 13 |  |  |
|  | 1-3 times per month | | 0 | 4 |  |  |
|  | none | | 60 | 51 |  |  |
| corn, chestnut rice, millet | almost every day | | 3 | 1 | 1.661 | 0.646 |
|  | 1-3 times per week | | 1 | 2 |  |  |
|  | 1-3 times per month | | 1 | 2 |  |  |
|  | none | | 85 | 82 |  |  |
| vegetables | almost every day | | 5 | 5 | 2.510 | 0.473 |
|  | 1-3 times per week | | 10 | 15 |  |  |
|  | 1-3 times per month | | 0 | 1 |  |  |
|  | none | | 75 | 66 |  |  |
| fruits | almost every day | | 3 | 3 | 1.484 | 0.686 |
|  | 1-3 times per week | | 12 | 12 |  |  |
|  | 1-3 times per month | | 2 | 5 |  |  |
|  | none | | 73 | 67 |  |  |
| meat | almost every day | | 2 | 0 | 5.799 | 0.122 |
|  | 1-3 times per week | | 1 | 5 |  |  |
|  | 1-3 times per month | | 0 | 1 |  |  |
|  | none | 87 | | 81 |  |  |
| liver and other animal viscera | almost every day | 1 | | 0 | 4.139 | 0.247 |
|  | 1-3 times per week | 1 | | 2 |  |  |
|  | 1-3 times per month | 0 | | 3 |  |  |
|  | none | 88 | | 82 |  |  |
| fish, shrimp, and crab | almost every day | 1 | | 0 | 0.967 | 0.617 |
|  | 1-3 times per week | 0 | | 0 |  |  |
|  | 1-3 times per month | 1 | | 1 |  |  |
|  | none | 88 | | 86 |  |  |
| egg | almost every day | 2 | | 1 | 0.852 | 0.837 |
|  | 1-3 times per week | 3 | | 4 |  |  |
|  | 1-3 times per month | 1 | | 2 |  |  |
|  | none | 84 | | 80 |  |  |
| cheese | almost every day | 1 | | 0 | 0.967 | 0.326 |
|  | 1-3 times per week | 0 | | 0 |  |  |
|  | 1-3 times per month | 0 | | 0 |  |  |
|  | none | 89 | | 87 |  |  |
| nut | almost every day | 0 | | 0 | NA | 1.000 |
|  | 1-3 times per week | 0 | | 0 |  |  |
|  | 1-3 times per month | 0 | | 0 |  |  |
|  | none | 90 | | 87 |  |  |
| soy or legume products | almost every day | 1 | | 0 | 1.990 | 0.370 |
|  | 1-3 times per week | 0 | | 0 |  |  |
|  | 1-3 times per month | 0 | | 1 |  |  |
|  | none | 89 | | 86 |  |  |
| iron | none | 84 | | 78 | 5.541 | 0.063 |
|  | occasionally | 2 | | 8 |  |  |
|  | often | 4 | | 1 |  |  |
| zinc | none | 81 | | 80 | 0.287 | 0.866 |
|  | occasionally | 7 | | 5 |  |  |
|  | often | 2 | | 2 |  |  |
| calcium | none | 75 | | 75 | 0.421 | 0.810 |
|  | occasionally | 4 | | 4 |  |  |
|  | often | 11 | | 8 |  |  |
| Vitamin A, D | none | 10 | | 8 | 0.575 | 0.750 |
|  | occasionally | 6 | | 4 |  |  |
|  | often | 74 | | 75 |  |  |
| nitrogen compounds | none | 84 | | 80 | 0.157 | 0.925 |
|  | occasionally | 2 | | 2 |  |  |
|  | often | 4 | | 5 |  |  |
| unsaturated fatty acid | none | 78 | | 73 | 2.715 | 0.257 |
|  | occasionally | 4 | | 9 |  |  |
|  | often | 8 | | 5 |  |  |
| Take probiotics in the past 1 month | no | 90 | | 85 | -1.442 | 0.149 |
|  | yes | 0 | | 2 |  |  |
| Cephalosporins exposure | | | | | | |
| obstetric exposure | no | 66 | | 58 | -0.965 | 0.334 |
|  | yes | 24 | | 29 |  |  |
| obstetric exposure dose | none | 66 | | 58 | -0.997 | 0.319 |
|  | 1 IU | 23 | | 27 |  |  |
|  | 1.5 IU | 1 | | 2 |  |  |
| obstetric exposure interval (average, h) | | 1.41 | | 2.04 | -1.123 | 0.261 |
| 0-7 day of age exposure | no | 84 | | 82 | -1.342 | 0.179 |
|  | yes | 6 | | 5 |  |  |
| 0-1 months of age exposure | no | 81 | | 69 | -1.972 | 0.049 |
|  | yes | 9 | | 18 |  |  |
| 1-2 months of age exposure | no | 84 | | 79 | -0.621 | 0.534 |
|  | yes | 6 | | 8 |  |  |
| 2-6 months of age exposure | no | 77 | | 81 | -1.617 | 0.106 |
|  | yes | 13 | | 6 |  |  |
| reasons for cephalosporins exposure during 0-6 months of age | fever | 3 | | 7 | -1.354 | 0.176 |
|  | cough | 13 | | 14 | -0.304 | 0.761 |
|  | vomit | 0 | | 1 | -1.017 | 0.309 |
|  | diarrhea | 3 | | 6 | -1.076 | 0.282 |
|  | other/unknown | 10 | | 11 | -0.314 | 0.753 |

Note: The Mann-Whitney U test was used for binary categorical variables and numeric variable, and the Kruskal-Wallis test was used for multicategorical variables. The data presented in the "Birth characteristic" section are the results of multivariate regression analysis. P>0.05 indicates no statistical difference between the two groups
